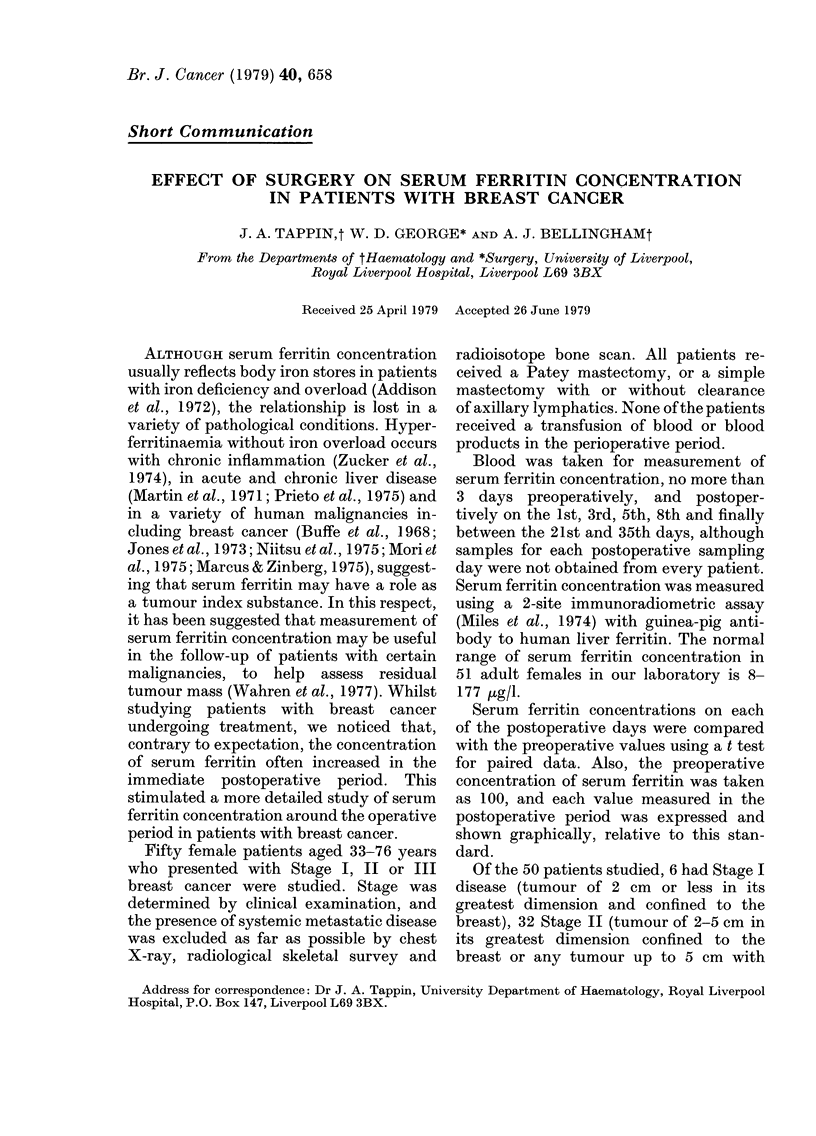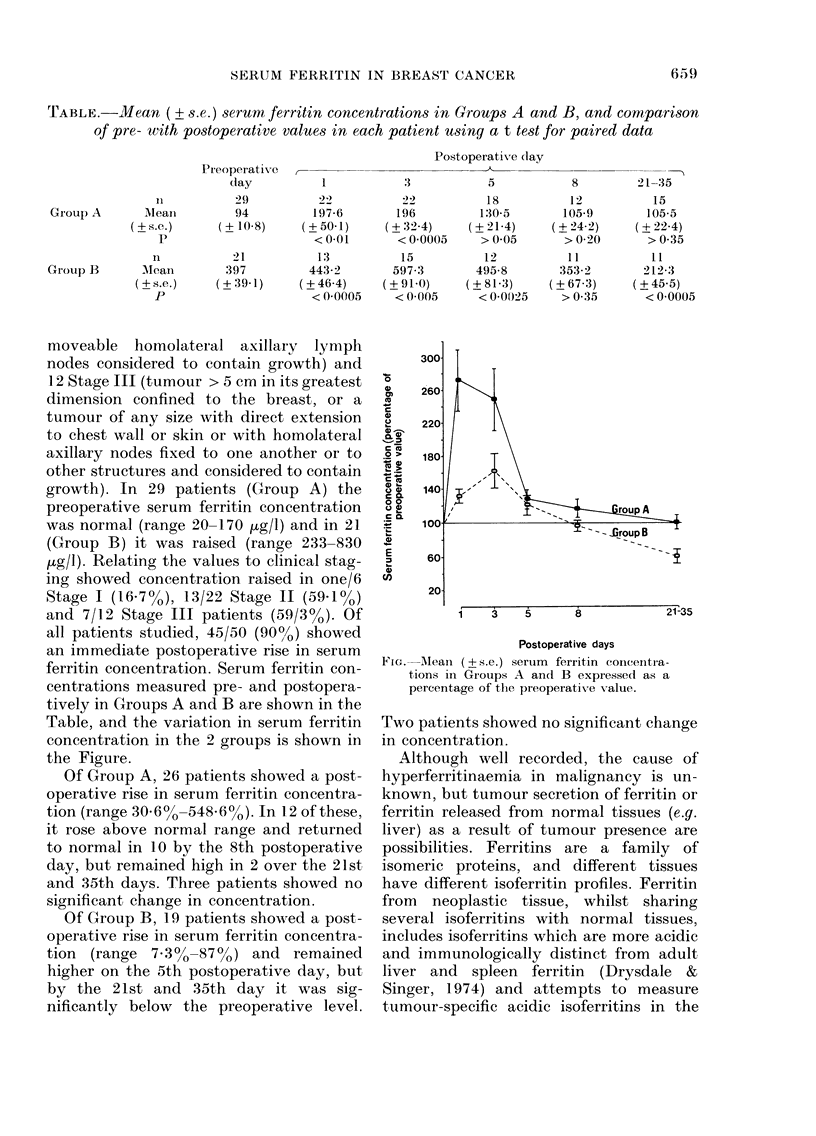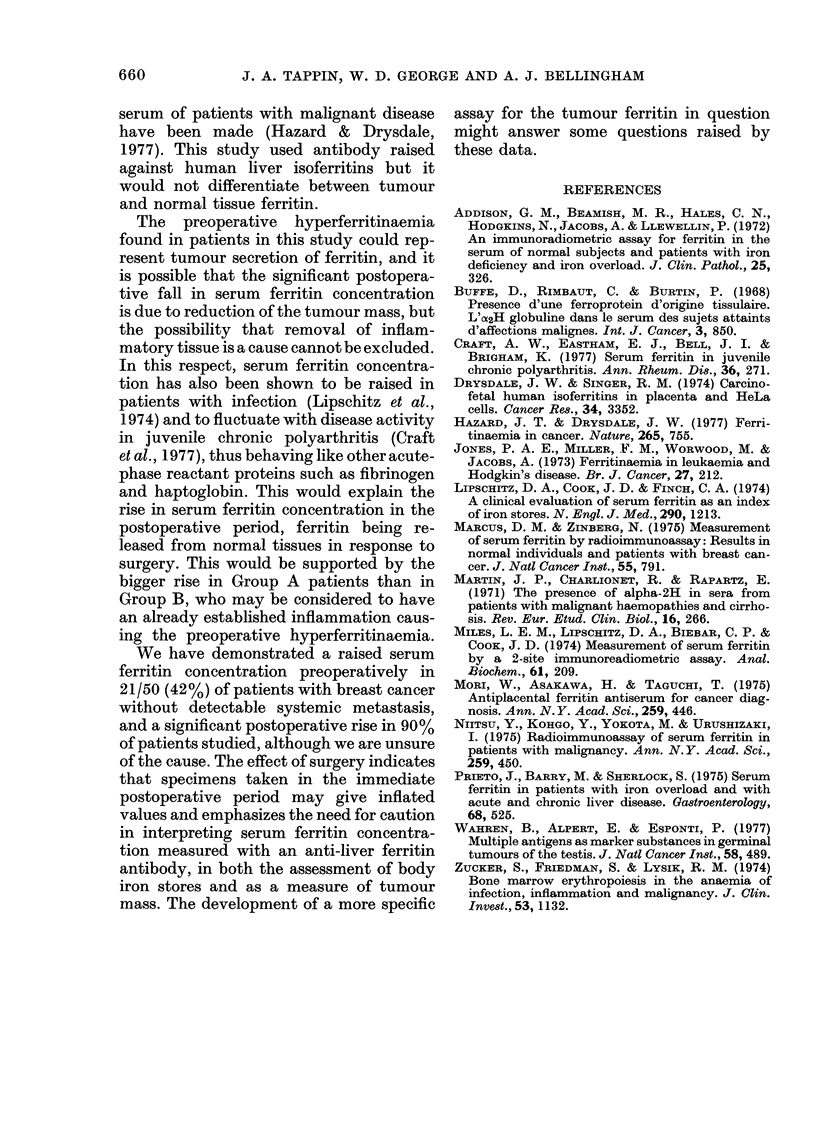# Effect of surgery on serum ferritin concentration in patients with breast cancer.

**DOI:** 10.1038/bjc.1979.232

**Published:** 1979-10

**Authors:** J. A. Tappin, W. D. George, A. J. Bellingham


*Br. J. Cancer* (1979) **40**, 658

***Short Communication***

### EFFECT OF SURGERY ON SERUM FERRITIN CONCENTRATION IN PATIENTS WITH BREAST CANCER

J. A. TAPPIN,† W. D. GEORGE* AND A. J. BELLINGHAM†

*From the Departments of †Haematology and *Surgery, University of Liverpool, Royal Liverpool Hospital, Liverpool L69 3BX*

Received 25 April 1979 Accepted 26 June 1979

ALTHOUGH serum ferritin concentration usually reflects body iron stores in patients with iron deficiency and overload (Addison *et al.*, 1972), the relationship is lost in a variety of pathological conditions. Hyperferritinaemia without iron overload occurs with chronic inflammation (Zucker *et al.*, 1974), in acute and chronic liver disease (Martin *et al.*, 1971; Prieto *et al.*, 1975) and in a variety of human malignancies including breast cancer (Buffe *et al.*, 1968; Jones *et al.*, 1973; Niitsu *et al.*, 1975; Mori *et al.*, 1975; Marcus & Zinberg, 1975), suggesting that serum ferritin may have a role as a tumour index substance. In this respect, it has been suggested that measurement of serum ferritin concentration may be useful in the follow-up of patients with certain malignancies, to help assess residual tumour mass (Wahren *et al.*, 1977). Whilst studying patients with breast cancer undergoing treatment, we noticed that, contrary to expectation, the concentration of serum ferritin often increased in the immediate postoperative period. This stimulated a more detailed study of serum ferritin concentration around the operative period in patients with breast cancer.

Fifty female patients aged 33–76 years who presented with Stage I, II or III breast cancer were studied. Stage was determined by clinical examination, and the presence of systemic metastatic disease was excluded as far as possible by chest X-ray, radiological skeletal survey and radioisotope bone scan. All patients received a Patey mastectomy, or a simple mastectomy with or without clearance of axillary lymphatics. None of the patients received a transfusion of blood or blood products in the perioperative period.

Blood was taken for measurement of serum ferritin concentration, no more than 3 days preoperatively, and postopertively on the 1st, 3rd, 5th, 8th and finally between the 21st and 35th days, although samples for each postoperative sampling day were not obtained from every patient. Serum ferritin concentration was measured using a 2-site immunoradiometric assay (Miles *et al.*, 1974) with guinea-pig antibody to human liver ferritin. The normal range of serum ferritin concentration in 51 adult females in our laboratory is 8–177 μg/l.

Serum ferritin concentrations on each of the postoperative days were compared with the preoperative values using a *t* test for paired data. Also, the preoperative concentration of serum ferritin was taken as 100, and each value measured in the postoperative period was expressed and shown graphically, relative to this standard.

Of the 50 patients studied, 6 had Stage I disease (tumour of 2 cm or less in its greatest dimension and confined to the breast), 32 Stage II (tumour of 2–5 cm in its greatest dimension confined to the breast or any tumour up to 5 cm with

Address for correspondence: Dr J. A. Tappin, University Department of Haematology, Royal Liverpool Hospital, P.O. Box 147, Liverpool L69 3BX.

TABLE.—*Mean ($\pm$ s.e.) serum ferritin concentrations in Groups A and B, and comparison of pre- with postoperative values in each patient using a* t *test for paired data*

| | | Preoperative day | Postoperative day 1 | 3 | 5 | 8 | 21–35 |
|---|---|---|---|---|---|---|---|
| | n | 29 | 22 | 22 | 18 | 12 | 15 |
| Group A | Mean | 94 | 197·6 | 196 | 130·5 | 105·9 | 105·5 |
| | ($\pm$s.e.) | ($\pm$10·8) | ($\pm$50·1) | ($\pm$32·4) | ($\pm$21·4) | ($\pm$24·2) | ($\pm$22·4) |
| | P | | $<0·01$ | $<0·0005$ | $>0·05$ | $>0·20$ | $>0·35$ |
| | n | 21 | 13 | 15 | 12 | 11 | 11 |
| Group B | Mean | 397 | 443·2 | 597·3 | 495·8 | 353·2 | 212·3 |
| | ($\pm$s.e.) | ($\pm$39·1) | ($\pm$46·4) | ($\pm$91·0) | ($\pm$81·3) | ($\pm$67·3) | ($\pm$45·5) |
| | *P* | | $<0·0005$ | $<0·005$ | $<0·0025$ | $>0·35$ | $<0·0005$ |

moveable homolateral axillary lymph nodes considered to contain growth) and 12 Stage III (tumour > 5 cm in its greatest dimension confined to the breast, or a tumour of any size with direct extension to chest wall or skin or with homolateral axillary nodes fixed to one another or to other structures and considered to contain growth). In 29 patients (Group A) the preoperative serum ferritin concentration was normal (range 20–170 $\mu$g/l) and in 21 (Group B) it was raised (range 233–830 $\mu$g/l). Relating the values to clinical staging showed concentration raised in one/6 Stage I (16·7%), 13/22 Stage II (59·1%) and 7/12 Stage III patients (59/3%). Of all patients studied, 45/50 (90%) showed an immediate postoperative rise in serum ferritin concentration. Serum ferritin concentrations measured pre- and postoperatively in Groups A and B are shown in the Table, and the variation in serum ferritin concentration in the 2 groups is shown in the Figure.

Of Group A, 26 patients showed a postoperative rise in serum ferritin concentration (range 30·6%–548·6%). In 12 of these, it rose above normal range and returned to normal in 10 by the 8th postoperative day, but remained high in 2 over the 21st and 35th days. Three patients showed no significant change in concentration.

Of Group B, 19 patients showed a postoperative rise in serum ferritin concentration (range 7·3%–87%) and remained higher on the 5th postoperative day, but by the 21st and 35th day it was significantly below the preoperative level.

FIG.—Mean ($\pm$s.e.) serum ferritin concentrations in Groups A and B expressed as a percentage of the preoperative value.

Two patients showed no significant change in concentration.

Although well recorded, the cause of hyperferritinaemia in malignancy is unknown, but tumour secretion of ferritin or ferritin released from normal tissues (*e.g.* liver) as a result of tumour presence are possibilities. Ferritins are a family of isomeric proteins, and different tissues have different isoferritin profiles. Ferritin from neoplastic tissue, whilst sharing several isoferritins with normal tissues, includes isoferritins which are more acidic and immunologically distinct from adult liver and spleen ferritin (Drysdale & Singer, 1974) and attempts to measure tumour-specific acidic isoferritins in the serum of patients with malignant disease have been made (Hazard & Drysdale, 1977). This study used antibody raised against human liver isoferritins but it would not differentiate between tumour and normal tissue ferritin.

The preoperative hyperferritinaemia found in patients in this study could represent tumour secretion of ferritin, and it is possible that the significant postoperative fall in serum ferritin concentration is due to reduction of the tumour mass, but the possibility that removal of inflammatory tissue is a cause cannot be excluded. In this respect, serum ferritin concentration has also been shown to be raised in patients with infection (Lipschitz *et al.*, 1974) and to fluctuate with disease activity in juvenile chronic polyarthritis (Craft *et al.*, 1977), thus behaving like other acute-phase reactant proteins such as fibrinogen and haptoglobin. This would explain the rise in serum ferritin concentration in the postoperative period, ferritin being released from normal tissues in response to surgery. This would be supported by the bigger rise in Group A patients than in Group B, who may be considered to have an already established inflammation causing the preoperative hyperferritinaemia.

We have demonstrated a raised serum ferritin concentration preoperatively in 21/50 (42%) of patients with breast cancer without detectable systemic metastasis, and a significant postoperative rise in 90% of patients studied, although we are unsure of the cause. The effect of surgery indicates that specimens taken in the immediate postoperative period may give inflated values and emphasizes the need for caution in interpreting serum ferritin concentration measured with an anti-liver ferritin antibody, in both the assessment of body iron stores and as a measure of tumour mass. The development of a more specific assay for the tumour ferritin in question might answer some questions raised by these data.

### REFERENCES

ADDISON, G. M., BEAMISH, M. R., HALES, C. N., HODGKINS, N., JACOBS, A. & LLEWELLIN, P. (1972) An immunoradiometric assay for ferritin in the serum of normal subjects and patients with iron deficiency and iron overload. *J. Clin. Pathol.*, **25**, 326.

BUFFE, D., RIMBAUT, C. & BURTIN, P. (1968) Presence d'une ferroprotein d'origine tissulaire. L'$\alpha_2$H globuline dans le serum des sujets attaints d'affections malignes. *Int. J. Cancer*, **3**, 850.

CRAFT, A. W., EASTHAM, E. J., BELL, J. I. & BRIGHAM, K. (1977) Serum ferritin in juvenile chronic polyarthritis. *Ann. Rheum. Dis.*, **36**, 271.

DRYSDALE, J. W. & SINGER, R. M. (1974) Carcinofetal human isoferritins in placenta and HeLa cells. *Cancer Res.*, **34**, 3352.

HAZARD, J. T. & DRYSDALE, J. W. (1977) Ferritinaemia in cancer. *Nature*, **265**, 755.

JONES, P. A. E., MILLER, F. M., WORWOOD, M. & JACOBS, A. (1973) Ferritinaemia in leukaemia and Hodgkin's disease. *Br. J. Cancer*, **27**, 212.

LIPSCHITZ, D. A., COOK, J. D. & FINCH, C. A. (1974) A clinical evaluation of serum ferritin as an index of iron stores. *N. Engl. J. Med.*, **290**, 1213.

MARCUS, D. M. & ZINBERG, N. (1975) Measurement of serum ferritin by radioimmunoassay: Results in normal individuals and patients with breast cancer. *J. Natl Cancer Inst.*, **55**, 791.

MARTIN, J. P., CHARLIONET, R. & RAPARTZ, E. (1971) The presence of alpha-2H in sera from patients with malignant haemopathies and cirrhosis. *Rev. Eur. Etud. Clin. Biol.*, **16**, 266.

MILES, L. E. M., LIPSCHITZ, D. A., BIEBAR, C. P. & COOK, J. D. (1974) Measurement of serum ferritin by a 2-site immunoreadiometric assay. *Anal. Biochem.*, **61**, 209.

MORI, W., ASAKAWA, H. & TAGUCHI, T. (1975) Antiplacental ferritin antiserum for cancer diagnosis. *Ann. N.Y. Acad. Sci.*, **259**, 446.

NIITSU, Y., KOHGO, Y., YOKOTA, M. & URUSHIZAKI, I. (1975) Radioimmunoassay of serum ferritin in patients with malignancy. *Ann. N.Y. Acad. Sci.*, **259**, 450.

PRIETO, J., BARRY, M. & SHERLOCK, S. (1975) Serum ferritin in patients with iron overload and with acute and chronic liver disease. *Gastroenterology*, **68**, 525.

WAHREN, B., ALPERT, E. & ESPONTI, P. (1977) Multiple antigens as marker substances in germinal tumours of the testis. *J. Natl Cancer Inst.*, **58**, 489.

ZUCKER, S., FRIEDMAN, S. & LYSIK, R. M. (1974) Bone marrow erythropoiesis in the anaemia of infection, inflammation and malignancy. *J. Clin. Invest.*, **53**, 1132.